# The bat–bird–bug battle: daily flight activity of insects and their predators over a rice field revealed by high-resolution Scheimpflug Lidar

**DOI:** 10.1098/rsos.172303

**Published:** 2018-04-04

**Authors:** Elin Malmqvist, Samuel Jansson, Shiming Zhu, Wansha Li, Katarina Svanberg, Sune Svanberg, Jens Rydell, Ziwei Song, Joakim Bood, Mikkel Brydegaard, Susanne Åkesson

**Affiliations:** 1 Lund Laser Centre, Department of Physics, Lund University, SE-22100 Lund, Sweden; 2 Center for Optical and Electromagnetic Research, South China Academy of Advanced Optoelectronics, South China Normal University, Guangzhou 510006, People's Republic of China; 3 Centre for Animal Movement Research, Department of Biology, Lund University, SE-22362 Lund, Sweden; 4 Guangdong Provincial Key Laboratory of High Technology for Plant Protection/Plant Protection Research Institute, Guangdong Academy of Agricultural Sciences, 7, Jinying Road, Tianhe District, Guangzhou 510640, People's Republic of China

**Keywords:** agriculture, bats, China, ecosystem service, entomology, predation

## Abstract

We present the results of, to our knowledge, the first Lidar study applied to continuous and simultaneous monitoring of aerial insects, bats and birds. It illustrates how common patterns of flight activity, e.g. insect swarming around twilight, depend on predation risk and other constraints acting on the faunal components. Flight activity was monitored over a rice field in China during one week in July 2016, using a high-resolution Scheimpflug Lidar system. The monitored Lidar transect was about 520 m long and covered approximately 2.5 m^3^. The observed biomass spectrum was bimodal, and targets were separated into insects and vertebrates in a categorization supported by visual observations. Peak flight activity occurred at dusk and dawn, with a 37 min time difference between the bat and insect peaks. Hence, bats started to feed in declining insect activity after dusk and stopped before the rise in activity before dawn. A similar time difference between insects and birds may have occurred, but it was not obvious, perhaps because birds were relatively scarce. Our observations are consistent with the hypothesis that flight activity of bats is constrained by predation in bright light, and that crepuscular insects exploit this constraint by swarming near to sunset/sunrise to minimize predation from bats.

## 1. Introduction

Many insects show either diurnal or nocturnal patterns of activity associated with physiological traits such as, e.g. thermoregulatory and/or predator defence adaptations [1,2]. However, there are also insects that seem to lack such defensive traits and rather minimize the predation risk by concentrating their flight activity to short swarming periods around dusk and dawn [3,4]. By adapting the latter strategy, they may employ predator satiation or selfish herd behaviour to reduce the predation risk at the individual level [5,6]. Such crepuscular insects are often the most prominent [7], and typically include many species of flies (Diptera) such as mosquitoes (Culicidae) [8] and midges (Chironomidae and Ceratopogonidae) [4,9], but also many moths (Lepidoptera) [10,11] and bugs (Hemiptera). The latter two categories include several serious pest species causing severe problems in rice fields [12].

Nevertheless, swarming and crepuscular insects are eaten by many types of predators, including aerial-hawking birds such as swallows and swifts [13–15], and many species of bats [16,17]. Hypothetically, the dusk insect activity peak represents a behavioural response to various constraints acting on the predators at this time of the day. For example, the feeding efficiency of birds is constrained by declining visual capacity at dusk [18,19], while the feeding activity of bats, which rely on echolocation, is believed to be constrained by predation pressure from raptorial birds in bright light [20–23]. Predation is a very strong evolutionary force, and it seems plausible that the entire crepuscular pattern of flight activity is influenced by predation, acting on the various components of the system, as outlined above. However, detailed studies of this phenomenon, which may be quite spectacular, have been limited methodologically because insects, birds and bats require different observation or monitoring methods, such as, for example, various insect traps, binoculars and ultrasound detectors [24]. Counts of individuals or estimates of flight activity are subject to different biases inherent in the various methods and are, therefore, hard to compare across groups.

A Lidar system for field observations of animal movements was recently developed [25,26], providing a powerful tool to investigate flight activity patterns in the field [27,28]. The device is able to overcome the problems mentioned above and may permit simultaneous monitoring of the activity of insects, birds and bats with high resolution in time and space [29], thus providing a relatively unbiased comparison of the activity of the different groups simultaneously and *in situ* over an extended period. We tested this system in an area of intensive agriculture (mostly rice fields) in southern China during the onset of the monsoon season in July 2016.

We examined the flight activity of the different groups of aerial fauna and its daily variation over the fields in accordance with the ideas outlined above. Specifically, we examined the following hypotheses: (i) foraging activity of birds and bats are constrained at dusk and dawn by decreasing light intensity and increasing predation risk, respectively, and (ii) crepuscular insects allocate the flight activity to dusk and dawn, when predation pressure may be relatively low owing to the constraints acting on the predators.

## 2. Material and methods

### 2.1. Study site and data collection

The field measurements were made over an experimental area at the Plant Protection Research Institute, Guangdong Academy of Agricultural Sciences (GAAS), just north of the city of Guangzhou in China (29 m above sea level; Lidar system at 23°23′31.11^″^ N, 113°25′30.40^″^ E) between 8 and 13 July 2016. The subtropical setting near the Tropic of Cancer means that the southwest Monsoon yields high precipitation and temperatures during the summer months. The surrounding area is dominated by intensive agriculture, notably rice *Oryza sativa* Linné, and urban or suburban environments.

**Figure 1.**
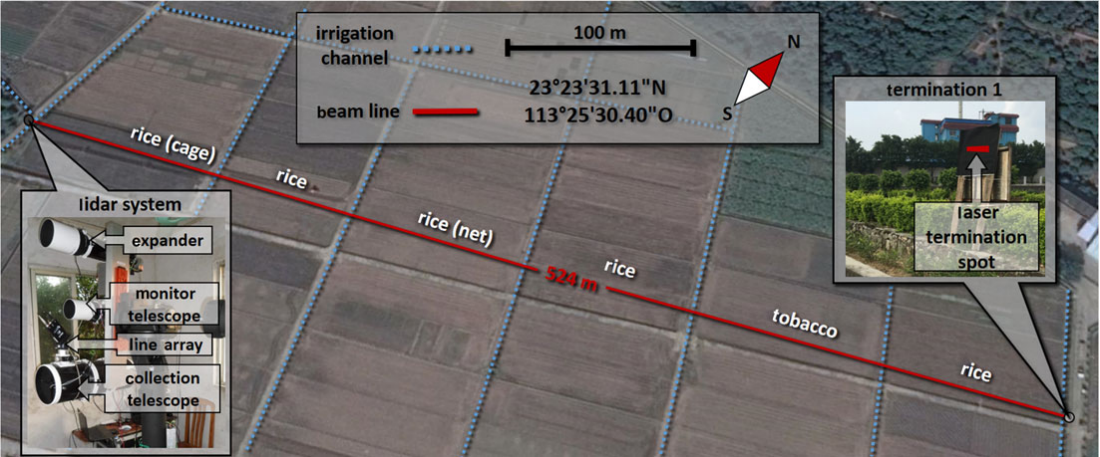
An aerial overview of the Lidar transect at the experimental farm in Guangzhou, China. The left insert shows the Lidar system and the right insert displays the laser termination with the laser spot marked in red.

The Lidar transect was static and covered the same horizontal air volume throughout the field campaign. During the campaign, the beam was emitted at approximately 4 m above the ground and crossed several rice fields and one field with tobacco *Nicotiana tabacum* Linné. The crops were at different stages of maturity in the different fields (figure 1). The beam was terminated on a board coated with black neoprene foam at 520 m range. The total probe volume, which is determined by the laser beam and pixel foot print, was about 2.5 m^3^ in our experiment. More information about how the size of the probe volume is determined can be found in [26]. We measured temperature and humidity continuously with a weather station located near the Lidar system throughout the study period. During one week of observations, the system measured continuously and a large amount of raw data was obtained. Observations were extracted from the raw data, and parametrized by using an adaptive threshold algorithm similar to the one described previously [30]. For each observation, we generated time, range and target size by this process. Some observations of insect behaviour in relation to weather conditions at our Guangzhou study site have been reported previously [27].

### 2.2. Insect capture and visual observations of insects, birds and bats

To collect and identify the pest insects occurring above the rice fields, we set up a ultraviolet lamp and a metal halide lamp with an insect trapping container during the evenings of 13 and 18 July at a location 105 m from the Lidar system and close to its field of view. Later we counted and identified the trapped insects in the laboratory. The data analysed in this work were not collected on the same days as the traps were in use. Visual observation of the activity over the field was made more or less regularly for most of the time that the Lidar was active, and the type and time of observations were entered into a notebook. We did not attempt to catch or otherwise identify the species of birds and bats flying over the fields during the study period although we documented the activity on video in the falling light at dusk.

### 2.3. Description of the Lidar system

A refractor telescope (ø = 120 mm, *f* = 600 mm) was used to shape the light from two continuous-wave high power laser diodes (808 nm, 3 W) into a beam and horizontally transmit it through the atmosphere. A Newtonian telescope (ø = 200 mm, *f* = 800 mm) was used to collect the laser light, backscattered by the atmosphere and its constituents, and image it onto a complementary metal oxide semiconductor (CMOS) linear array detector with high sampling rate (up to 4 kHz). A picture of the system is displayed in the insert of figure 1. The baseline separation between transmitter and receiver was 814 mm and the range was determined through triangulation. More details can be found in recent publications [27,31,32].

With this kind of Lidar, range resolution is obtained by sharply imaging the laser beam onto a linear array of detector pixels. The optical system is arranged according to the Scheimpflug principle [33,34], which means that sharp imaging along the entire transect is achieved by tilting the detector so that the system can use a large aperture for efficient light collection. The same principle is often applied to photography and then referred to as tilt-shift photography [35]. In Schiempflug Lidar, each pixel along the line detector will monitor the backscattered light from a different section of the laser beam, and range resolution is thus obtained along the line of detector pixels. The range scale is nonlinear, and the pixels monitoring the beam close to the Lidar system observe a smaller air volume than those monitoring the beam further away.

During the measurements we used two identical but perpendicularly linearly polarized lasers. The two laser beams overlapped and the laser outputs were modulated on and off sequentially in synchronization with the camera exposure. This means that the first laser was on during one exposure, the second laser was on during the next exposure and both lasers were off during the third exposure to record the optical background and enable online background subtraction [31]. The full sampling rate of the camera during the measurements was 3 kHz, resulting in an effective sampling rate of 1 kHz per laser band. A linear polarization filter was placed in front of the detector in such a way that its transmission direction was aligned with the linear polarization direction of one of the lasers. The signal in one camera exposure thus consisted of backscattered light, which had kept its polarization (co-polarized). The signal in the next exposure consisted of backscattered light, which had changed it polarization (de-polarized), whereas the third exposure contained the background signal. In this work, only data from the co-polarized exposures have been analysed. The reason for this is that the spatial overlap between the two laser beams was not adequate enough to properly match the signals from the same observation, and the co-polarized signal is the strongest of the two. For more information about polarization Lidar or polarization in biophotonics; see [27,32,36].

**Figure 2.**
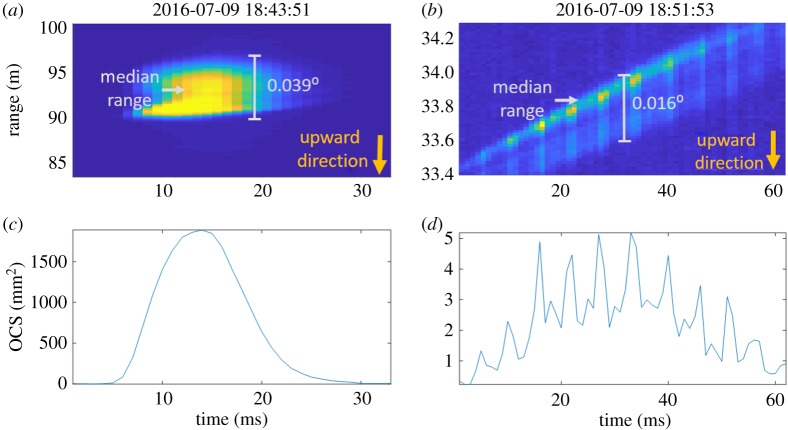
Examples of time-range maps of a large object observation (*a*) and a small object observation (*b*); (*c*) and (*d*) display the time series of (*a*) and (*b*), respectively. The observation of a small organism (presumably an insect) flying away from the detector displays a typical time modulation of the signal due to the wing beats. The observation of the larger organism in (*a*) does not display this kind of modulation because its wing beat frequency is too low to be resolved during its transect through the laser beam. In Scheimpflug Lidar range is determined from pixels at a specific observation angle, but the width of the beam induces a range uncertainty and observations of larger organisms will cover several pixels. The pixel interval can be represented as an opening angle for an observation. This corresponds to the difference in observation angle between its first and last pixel. The opening angle of each observation is marked in grey in (*a*) and (*b*). The opening angle and the median range are used to calculate the angular size of the observation. The wings of the organism give rise to a larger spread in the signal in the range direction when they are pointing up and down. The Lidar system monitored the laser beam from below. This means that when the wings of an organism are in a downward position the signal will be spread out towards ranges further away from the Lidar system and when they are in the upwards position the signal will be spread out towards closer ranges. The yellow arrows in the lower-right corners of the figures indicate the direction the signal will be spread out in when the wings of an organism are in the upwards direction. From this, it become clear that the large organism in (*a*) has the shape of a bat/bird with its wings in a downward position when it passes through the beam. It can also be concluded that the wing beats of the insect in (*b*) are visible when the wings are in the upwards position.

Typical signals from flying organisms as obtained with the Lidar are displayed in figure 2. One observation corresponds to a large organism (bird or bat; figure 2*a,c*), and one represents a small organism (insect; figure 2*b,d*). In the top panels, the observations are displayed in both time and space while the bottom panels show the time series of each observation. The signal intensity in these observations was calibrated to an optical cross section (OCS) during the data evaluation. OCS is a measurement of the optical size of an object, e.g. a flying organism, passing through the laser beam. It is the product of the cross-section area of the organism and its optical reflectivity (*R*). Since the distance to the termination screen is known, as well as the size of the laser spot on the termination and the reflectivity of the covering neoprene sheet, the OCS could be calibrated across the whole laser transect using the termination signal in each recorded file, as described elsewhere [28].

Owing to the finite width of the laser beam [26] and the fact that the laser beam is viewed at an angle in the Scheimpflug Lidar configuration, the signal from an observed organism will not only be detected in the pixel corresponding to its specific range, but it will be smeared out across several pixels. This can be seen in both observations in figure 2*a,b*. This spread of the signal in range can in fact be exploited to get another estimation of the size of an organism passing through the beam. By using the length of an observation on the detector and its range, the angular size of the organism across the beam can be obtained. This size is called the ‘apparent size’. It is important to point out that this quantity does not scale with reflectance. In figure 2*a,b*, the opening angle (due to the spread in range of the signal) and the median range of the observation are marked in grey. The apparent size of an observation is given by the median range multiplied by the tangent of the opening angle.

**Figure 3.**
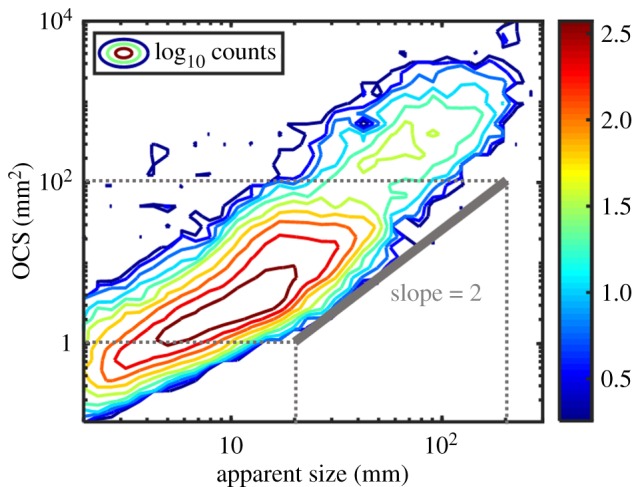
Contour plot of the OCS and the apparent size of the Lidar data evaluated in this paper. The grey line represents a slope of 2.

As OCS gives a measurement of the size as an area, and apparent size gives the size in height across the beam, the relationship between the terms has a quadratic dependence. In a double logarithmic plot where OCS is a function of apparent size, the slope should thus be two. Figure 3 presents a contour plot of the OCS and apparent size of the Lidar observations analysed in this work. The grey line in the plot corresponds to a slope of two. The slope of our observations is similar to the grey curve, indicating a correct size calibration.

### 2.4. Lidar observations and sensitivity of the Lidar system

In this work, the number of Lidar observations over time is presented, and this is used as an indication of the flight activity. This should, of course, not be confused with population sizes or number of individuals. One individual organism may give rise to several observations.

The sensitivity of the Lidar system will result in a minimum detectable size and this will change over time and range. The sensitivity is a function of both the optical background and the range (*r*). The minimum organism size that can be detected by the Lidar system increases with increasing range, owing to the decreasing signal strength of the backscattering at far distances. Small organisms are thus more likely to be detected close to the system than far away from it. However, this bias affects the absolute number of detected insects at each time, but not the relative number of detected insects over time, which is the aspect of interest in this investigation.

**Figure 4.**
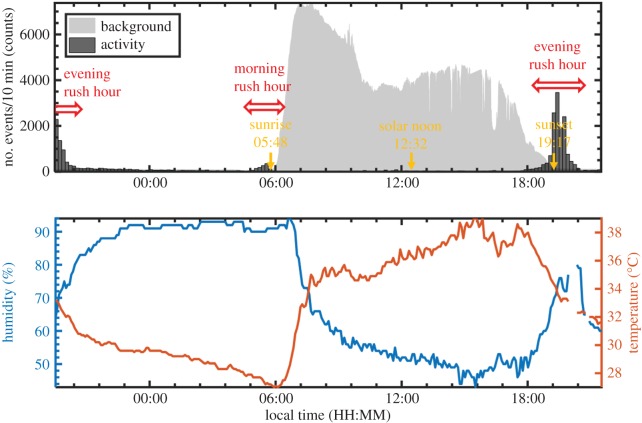
An overview of the activity (number of observations detected by the Lidar system, *n* = 27, 212), and the environmental conditions (temperature and humidity) prevailing on 8 and 9 July 2016. During dawn and dusk there was a clear increase in the number of observations, indicated as ‘rush hour’. The optical background level is shown in grey. The activity and background readings are shown on a linear scale in this case.

The optical background level is higher in daylight than during the night. This leads to higher noise level and as a result lower sensitivity of the system. The change in optical background can clearly be observed by looking at the grey area plot in figures 4, 6 and 7, which show how the detected optical background changes during the day. This means that smaller objects can be detected during the night than during the day. This bias may affect the detected distribution of smaller organisms (insects) over time, but not the distribution of the larger ones (birds and bats). A more detailed evaluation of this problem is provided in the electronic supplementary material.

## 3. Results

### 3.1. Daily flight activity

We obtained 2 × 10^5^ aerofauna observations during one week of fieldwork, and here we report on a subset of 41 797. These observations were all collected during three periods when the sampling was not interrupted by rainfall or technical issues, namely (i) 8 July 19.30 h to 9 July 22.30 h, (ii) 11 July 03.30–07.30 h and (iii) 11 July 17.30–21.30 h.

An overview of the flight activity (number of observations) in the probe volume during a continuous observation period (sample period (i)) is shown in figure 4 as an example of the kind of data obtained. The distribution shows distinct peaks at dawn and dusk with a higher one at dusk. The flight activity differed by one or even two orders of magnitude at dusk and dawn compared to the periods before and after (figure 4). The grey area plot in the figure displays the optical background signal (the signal during dark time slots) as an indicator of how the light level changes during the day. The background signal also affects the sensitivity of the Lidar system as discussed above and in the electronic supplementary material. The day of 9 July was very warm and with relatively low humidity, as shown in the lower panel in figure 4.

### 3.2. Birds, bats and insects

Very little bird activity was observed during the middle of the day, but the activity increased considerably during the twilight periods, when many birds such as swallows *Hirundo* spp. [37] flew in all directions, apparently hunting insects at low altitudes above the fields. The bats observed over the field visually at dusk and dawn frequently made chases and dives, as expected if they were actively hunting insects. There were several species of aerial-hawking bats, which differed considerably in size, and they occurred regularly from about 1 m above the ground to at least 10–20 m. Based on their appearance and flight style, the visually observed bats most likely included species of ‘house bats’ in the Genera *Pipistrellus* and *Scotophilus* and perhaps also other species [38,39]. During approximately 15 min around dusk and dawn, the activity of bats and birds overlapped, so that both were observed hunting over the field simultaneously, although in relatively low numbers.

Visual observations and videos taken in natural light indicated abundant small swarming flies, such as non-biting midges (Chironomidae), over the field at dusk and dawn. Our insect capture effort at night showed that several major insect pest species also were present over the fields, including the moths *Chilo suppressalis* Walker and *Spodoptera litura* Fabricius, which feed on rice and tobacco, respectively. There were also several species of bugs (Hemiptera), some of which are serious pests on rice, including brown plant hopper *Nilaparvata lugens* Stål, grey plant hopper *Laodelphus striatellus* Fallén, white-backed plant hopper *Sogatella furcifera* Horvath, white-winged leaf hopper *Thaia rubiginosa* Kuoh, zigzag-striped leaf hopper *Inazuma dorsalis* Motschulsky, black-tailed leaf hoppers *Nephotettix bipunctatus* Fabricius and *N. virescens* Distant and the coreid bug *Leptocorisa varicornis* Fabricius. In addition to these pest species, we captured several species of bugs, beetles, flies and ants that do not feed on rice or tobacco.

### 3.3. Size discrimination of the Lidar observations

**Figure 5.**
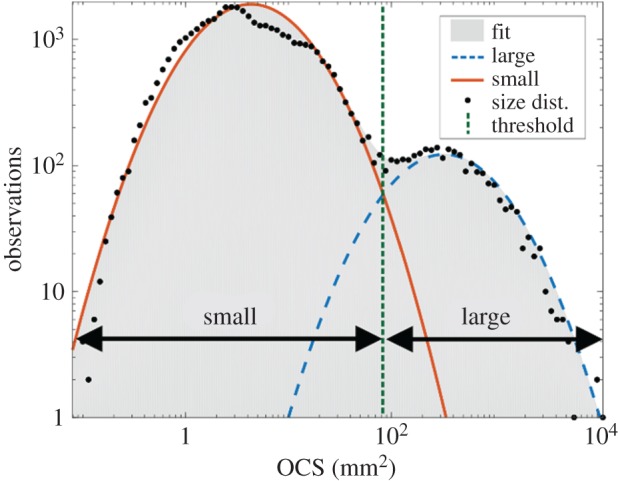
A histogram of the maximum OCS of each observation during two dusk and two dawn periods plotted on logarithmic scales. This size distribution has two distinct modes—one for small objects and one for large ones. Two parabolas were fitted to these modes. The intersection between the two fitted curves was used to set a size threshold for the discrimination between small and large targets. The resulting threshold is 84 mm^2^.

To discriminate between small and large objects, i.e. insects and birds/bats, respectively, a size threshold was set based on the size distribution, or the maximum OCS of each organism during its trajectory through the beam (*n* = 41 797; figure 5). This distribution shows a clear bimodality, and the boundary between the two modes is set as a size threshold (84 mm^2^). The threshold was found by fitting parabolas (Gaussians in logarithmic scale) to the two modes and finding their intersection. We emphasize that the method allowed separation of small and large organisms, but it was not possible to say whether a specific observation was a bat or a bird, so this distinction was based on the flight time in relation to sunset/sunrise for each group, which, fortunately, is well known. The feeding efficiency of birds such a swifts and swallows is limited by visual capacity at low light levels [18,19] when they ascend to higher altitudes [40], while aerial-hawking bats, including those observed in this study, usually emerge to feed within around 30 min after sunset [20,21].

**Figure 6.**
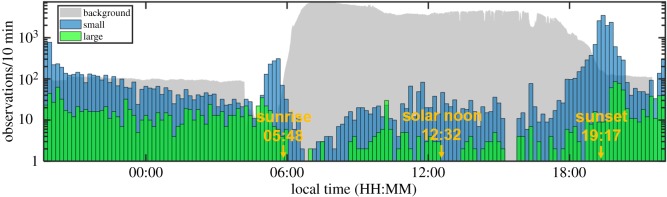
An overview of the activity during the same time-period as in figure 4, but with object size discrimination implemented. The *y*-axis has a logarithmic scale in this case. The grey area plot in the figure displays the optical background signal.

In figure 6, the same overview of the activity as in figure 4 is shown, but with the observations split into small and large objects. The activity of small objects (insects) was generally higher during the night than during the day, and a corresponding difference was recorded for their predators. Activity of large objects was in general much higher during the night (bats) than during the day (birds), differing by about an order of magnitude on average.

### 3.4. Dusk and dawn observations—rush hours

**Figure 7.**
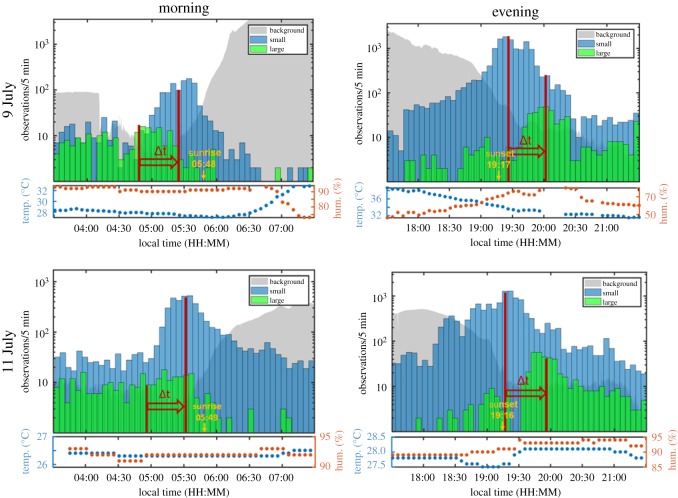
Observations per 5 min periods during dawn and dusk on 9 July and 11 July with target size discrimination implemented. The median of the activity distributions for the small (blue) and large (green) targets over the selected time windows were found as indicators of the position of peak activity. These are indicated with red lines. The difference in time (Δ*t*) is 36.7 min (s.d. = 1.3, *n* = 4). The grey area plot shows the optical background and it indicates the change in light level during the time periods.

The temporal distributions of both large and small objects show distinct peaks before sunrise and after sunset (figures 6 and 7). However, the peaks of the small and the large objects were well separated in time, with the small objects (insects) being most active before the majority of the larger ones (mostly bats) at dusk, and after most of them at dawn. This time separation was quantified using the median of the activity distributions in the selected time windows (03.30–07.30 h, 17.30–21.30 h). The difference in median value was remarkably similar for all four rush hour periods, 36.7 ± 1.3 min (figure 7).

The increasing bird activity at dawn to some extent overlapped temporally with the falling bat activity and vice versa. Birds and bats were typically observed over the fields simultaneously only during brief periods at dusk and dawn and then only in low numbers.

## 4. Discussion

The present study was primarily an evaluation of the performance of the new Lidar system under real-field conditions, to illustrate its possibilities and constraints. Clearly, the results that we obtained were detailed and met with our expectations, although the data were collected in just a few days. Nevertheless, the integrated and detailed observations of insects, birds and bats that we made during this study would not have been possible by use of any other method currently available [41]. For example, observing birds and bats with the same technique facilitated quantitative and relatively unbiased comparisons of the two groups at the same time, and, indeed, we were quite surprised to find that the bats, or at least their activity, outnumbered the birds by as much as an order of magnitude at our site. Of course, the generality of this observation cannot be evaluated without many more observations spread across seasons and habitats.

Several species of bats and birds exploit flying insects for food and the temporal occurrence of prey obviously has a strong influence on the foraging effort and timing of the predators. However, the predators also have to trade-off foraging efficiency and their own predation risk, and their foraging efforts may also influence the activity patterns of insect prey secondarily. For example, bats that feed on crepuscular insects typically emerge from their roost and start to feed while the peak activity of insects is already declining [42]. Although earlier emergence may have resulted in higher food intake, it may also have increased the predation risk in the brighter light [43,44]. Without the proposed predation constraint acting on the bats, the bat feeding activity would be expected to coincide entirely with the flight activity of the insects, assuming that the bats try to maximize their feeding efficiency. However, the mean time delay between the insect and bat activity peaks was as much as 37 min, suggesting that bat and insect activity did not coincide. The observed temporal discrepancy may be an effect of avoidance responses of the individual insects, i.e. allocation of the swarming activity to a period when feeding activity of bats is still relatively low. In addition, the density of aerial insects could eventually have been reduced by predation, as more and more bats started to feed. It seems possible that both mechanisms may have contributed to the observed pattern.

A similar situation could have applied to the birds, although this was not as obvious at our site, perhaps because of relatively low numbers of birds. The feeding by swallows and swifts is eventually hindered by the falling light at dusk (the opposite occurs at dawn) [19], and this will give the insects a chance to ‘escape’ predation by swarming when the light intensity is lower. This would be analogous to the bat avoidance response described above, and if the two act in concert, a sharp insect activity peak would be expected to occur when neither predator category is able to feed efficiently. However, in our case, the birds were relatively rare components of the aerial fauna, and the temporal discrepancy relative to the insect peak was not as obvious as for the bats (figure 6). Possibly, the insects' avoidance of bats was stronger and more consistent because the predation pressure from bats was higher. Alternatively, the insects disappeared faster owing to predation, basically for the same reason.

### 4.1. Is there a ‘predation-free time window’ at dusk?

**Figure 8.**
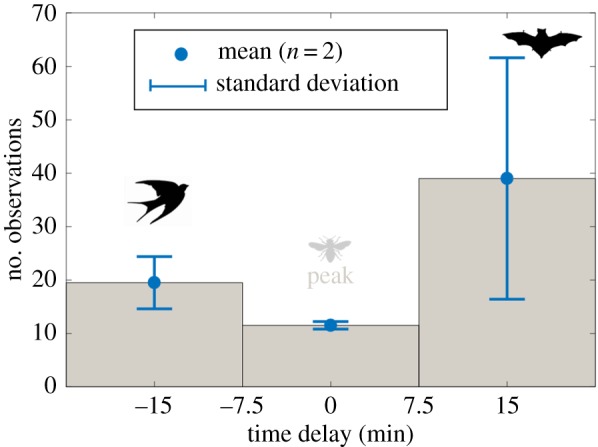
The number of observations of large objects (bats and birds) during the evening rush hours (*n* = 2). Δ*t* = 0 represents the median value of the activity distribution of small organisms (insects) at dusk.

If the feeding activity of bats and birds is constrained in bright and dim light, respectively, as outlined above, we may expect to find a period at dusk when neither of them feed efficiently, i.e. there would be a ‘predation-free time window’ or at least a ‘time window when predation risk is minimal’, and this window could then be exploited by insects [11,45]. Indeed, our observations indicate that the swarming insects separated temporally from bats by 37 min on average (figure 7). The separation could have resulted from a shift in the flight activity, whether the insects are also separated from birds remains unclear, however. Nevertheless, a closer look at the data indicate that such a window of relatively low predation pressure actually may have existed, although this is based on just the two evenings for which we obtained sufficient amounts of relevant data. In both cases, the peak activity of small objects (insects) coincided with dips in the activity of the large organisms (bats and birds), as expected. To illustrate this, the time-period around the peak insect activity was divided into three intervals, each with a length of 15 min; I before the peak, II encompassing the peak (±7.5 min around the median) and III after the peak (figure 8). Although the activity of bats and birds overlapped during approximately 15 min at dusk, the absolute number of observations of both groups taken together was at its lowest during this particular interval, when compared to the 15 min immediately before and after, when only one of the predatory categories was present.

In summary, the results speak in favour of an explanation to the dusk/dawn scenario according to our hypothesis. It is influenced by several ecological factors including the ambient light level, important for foraging in birds [18,19], predation pressure from raptorial birds on bats, essentially restricting bat activity to dark periods [20,21], and predation pressure from both birds and bats, either restricting the flight period of the insects and/or cropping them by intensive feeding. Hence, we find it possible that the timing of the swarming flights of some aerial insects has evolved in response to the short time window of minimal predation risk that arise near dusk and dawn as a secondary consequence of constraints acting on the predators.

### 4.2. Ecosystem services and other implications

By this work we aimed to understand a fundamental behavioural pattern in crepuscular insects and their predators, but the results may also have implications for agricultural management, including insect pest control, and, therefore, be worth further consideration. For example, it was quite surprising that bat activity was so high and persistent over the field, considering the setting in an intensively farmed semi-urban area. Aerial-hawking bats are opportunistic feeders and typically switch to whatever insects are available. They move over extensive areas and accumulate at outbreaks of pest insects [46]. This include the rice fields in southeast Asia and also a major pest species observed in this study, namely the brown plant hopper [47]. Aerial-hawking bats are extremely efficient foragers on flying insects, individuals maintaining feeding rates of 10 insects or more per minute over extended periods are common and may include typical capture success near 100% [48]. Furthermore, our observations show that high activity of bats over the rice fields was maintained consistently throughout the nights of observation (figure 6).

In comparison, the activity of birds was rather low and relatively inconsistent, but even so, predation on the pest insects presumably continued throughout the period of activity. However, the distribution of aerial insect prey in daytime may be different when compared to at night, and the distribution and foraging effort of aerial birds may have been directed towards other habitats [15,49].

The bugs observed in our study are important pests on rice, not only because they feed on it but also because they are vectors of viral infections [50]. Activity pattern of e.g. the brown plant hopper is usually crepuscular and bimodal with peaks at dusk and dawn [12], while the striped stem borer, a moth of the family Crambidae, which are equipped with ultrasonic ears that can detect bats, is more nocturnal [51]. Nevertheless, our observations indicate that crepuscular and nocturnal pests are both exposed to bat predation to a similar extent. For more specific information about the pest insects considered here, including their management and pesticide resistance, we refer to http://www.cabi.org/isc/datasheet/.

Insectivorous bats are able to control the growth of insect populations in agricultural ecosystems [52], with economic implications that should not be underestimated. For example, the annual worth of bats' pest control service for the United States agriculture is believed to be about 23 billion dollars [53]. The corresponding value of the bats' service for the rice production in China and the rest of eastern Asia has never been estimated, as far as we know, but is presumably even higher. The biological control and ecosystem service provided by bats (and birds) probably will become more and more important as pesticide resistance in bugs and other insects evolve and become more prevalent and as sustainable food production needs to be maintained. Accordingly, the continued welfare of the bat (and bird) populations in intensively managed agricultural systems like the one we studied may become critically important, and should always be considered seriously.

## Supplementary Material

Malmqvist_Text_ESM

## Supplementary Material

Malmqvist_Figure_i_ESM

## Supplementary Material

Malmqvist_Figure_ii_ESM

## Supplementary Material

Malmqvist_Table_i_ESM

## Supplementary Material

Malmqvist_ObservationData_ESM

## Data accessibility

Data can be found as a spread sheet in the electronic supplementary material.
