# Supplementary material for: The bat–bird–bug battle: daily flight activity of insects and their predators over a rice field revealed by high-resolution Scheimpflug Lidar
Source: R Soc Open Sci. 2018 Apr 4;5(4):172303. doi: 10.1098/rsos.172303 (PMC5936944; doi:10.1098/rsos.172303)
Supplement: Malmqvist_Text_ESM [file rsos172303supp1.pdf]

Supplementary material

The optical background level is higher during the day than during night-time. This can clearly be seen by looking at the grey area plot in figures 4, 6 and 7, which show how the detected optical background changes during the day. This leads to higher noise levels and, as a result, to lower sensitivity of the system. Consequently, the minimum detectable OCS will be lower during the night than during the day and some small objects that are detected during night-time will not be detected during the day. This bias may affect the detected distribution of smaller organism (insects) over time, but not the distribution of the larger ones (birds and bats).

To evaluate the effect that this bias may have on the shape of the activity distributions, the equivalents of figures 6 and 7 were plotted again, but with the threshold set to the highest minimum detectable OCS at each range during the time period in question. This corresponds to setting the sensitivity of the system to the lowest one observed during the time period in question. The result may be seen in figures i and ii. Table i summarizes the difference between the total number of observations and the number of observations when the maximum threshold is used for the 4 rush hours and also for the longer period (8-9 July, 19.30-22.30 h) which includes two of the rush hours. For the dusk and dawn periods the change in number of observations is less than 1%. Not surprisingly, the decrease for the longer time period, which includes both night and day, is much larger. Despite this, the shape of the distribution of the observations of small organisms does not change greatly during the periods in question.

Table i.

Table i. Summary of the difference in number of observations when the sensitivity of the system is taken into account.

	**Total # of observations**	**# of observation above threshold**	**Decrease in # of observations**
**9 July, 0330-0730h**	1532	1528	0.3%
**11 July, 0330-0730h**	5001	4995	0.1%
**9 July, 1730-2130h**	14544	14424	0.8%
**11 July, 1730-2130h**	12913	12903	0.08%
**8-9 July, 1930-2230h**	23883	20270	15%

Fig. i.

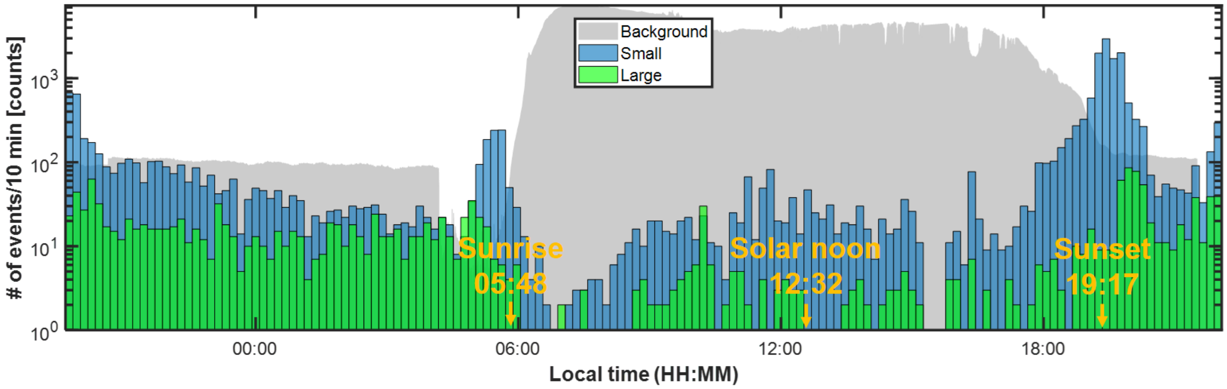

Figure i. Observations per 5 min during the first 27 h of the measurement campaign with target size discrimination implemented and the detection threshold set to the maximum for each range during the time period.

Fig. ii.

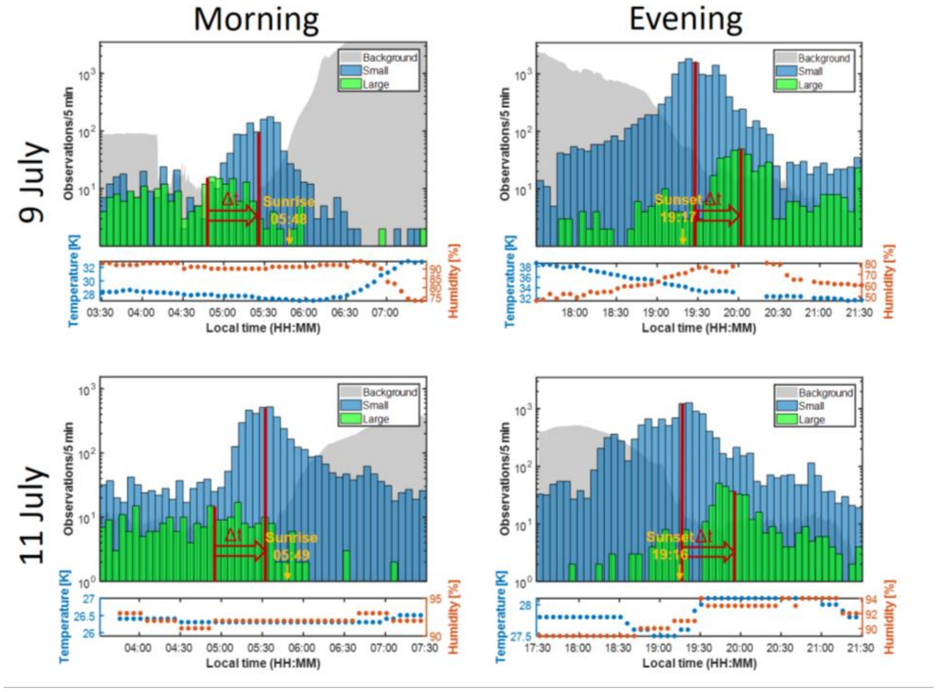

Figure ii. Observations per 5 min during dawn and dusk on 9 July and 11 July with target size discrimination implemented and the detection threshold set to the maximum for each range during the time period.
