# Supplementary figures and images for: The bat–bird–bug battle: daily flight activity of insects and their predators over a rice field revealed by high-resolution Scheimpflug Lidar
Source: R Soc Open Sci. 2018 Apr 4;5(4):172303. doi: 10.1098/rsos.172303 (PMC5936944; doi:10.1098/rsos.172303)

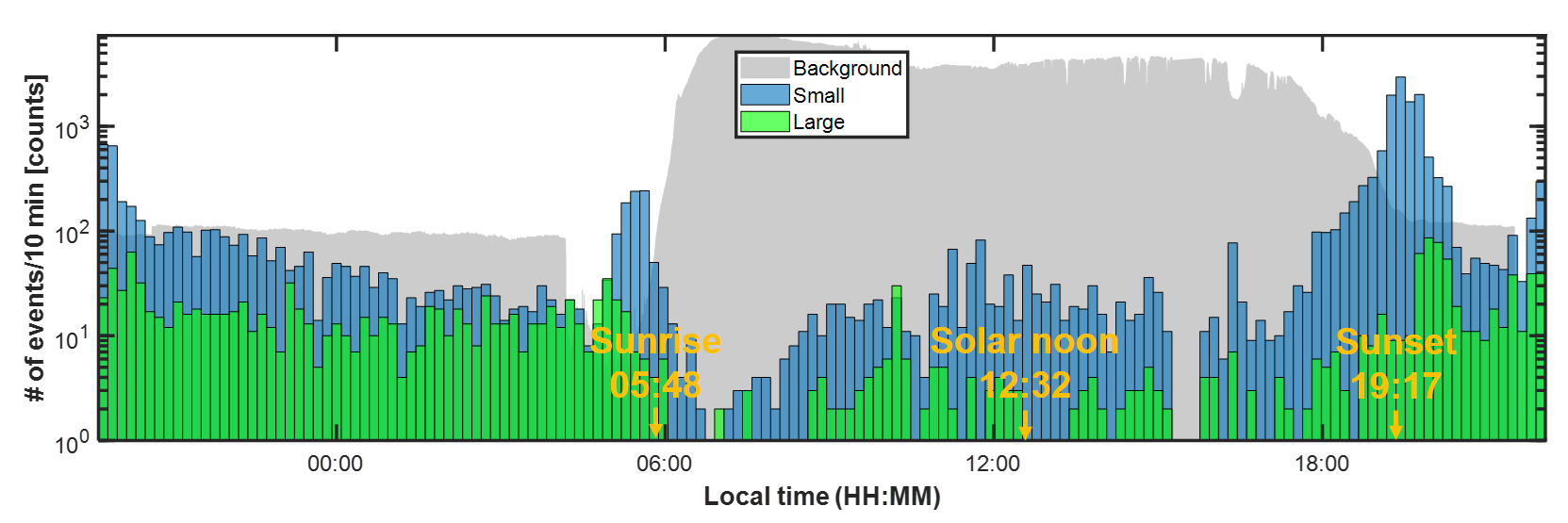

Supplement: Malmqvist_Figure_i_ESM [file rsos172303supp2.PNG]

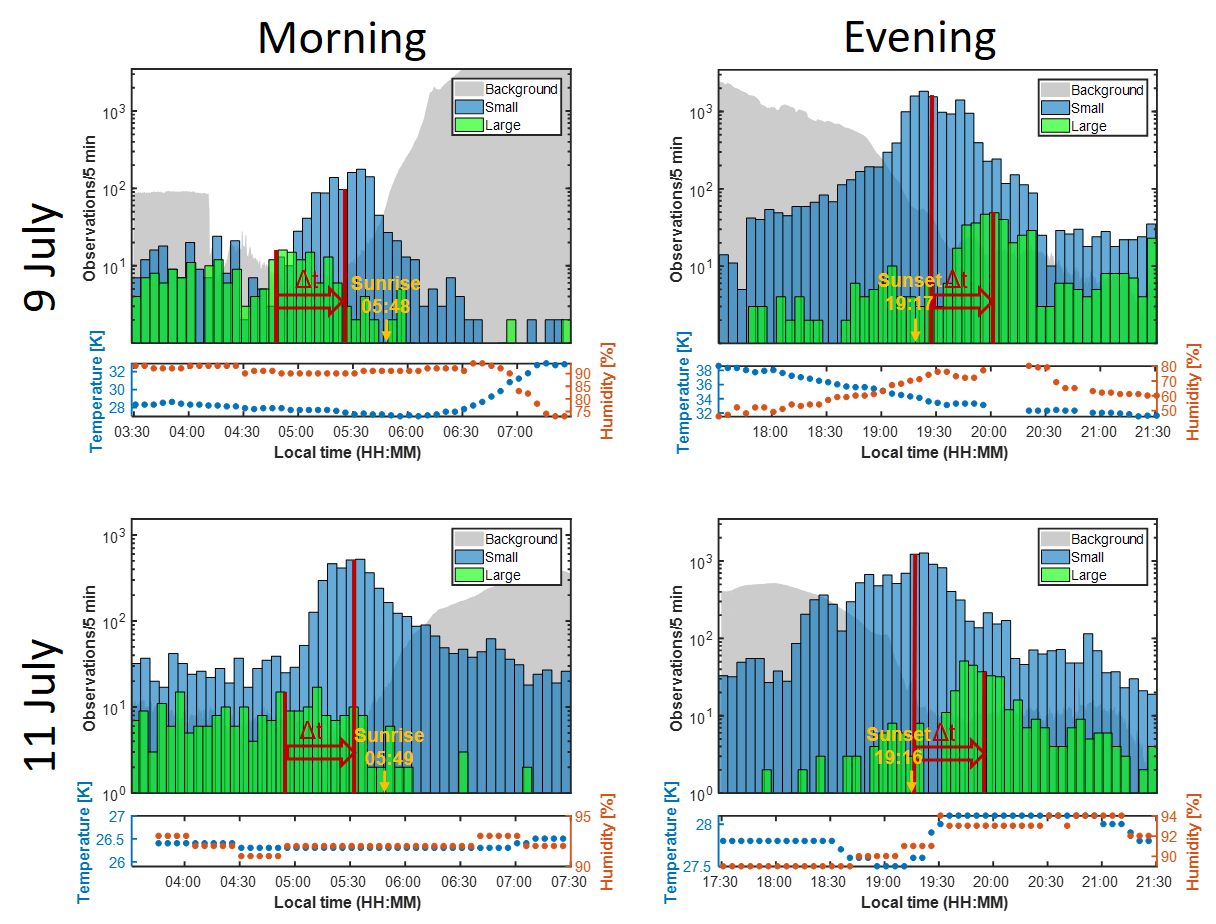

Supplement: Malmqvist_Figure_ii_ESM [file rsos172303supp3.PNG]

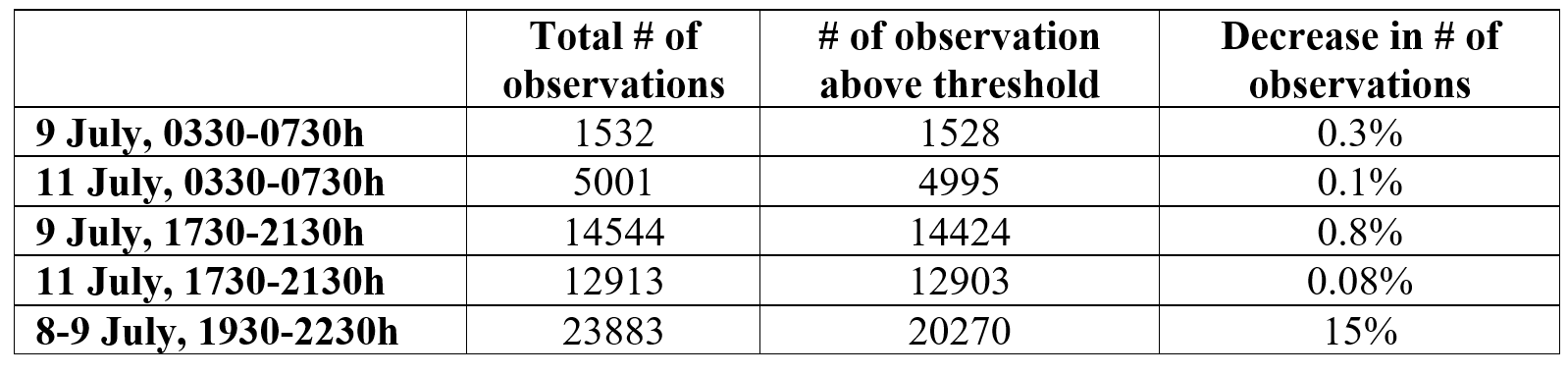

Supplement: Malmqvist_Table_i_ESM [file rsos172303supp4.PNG]
